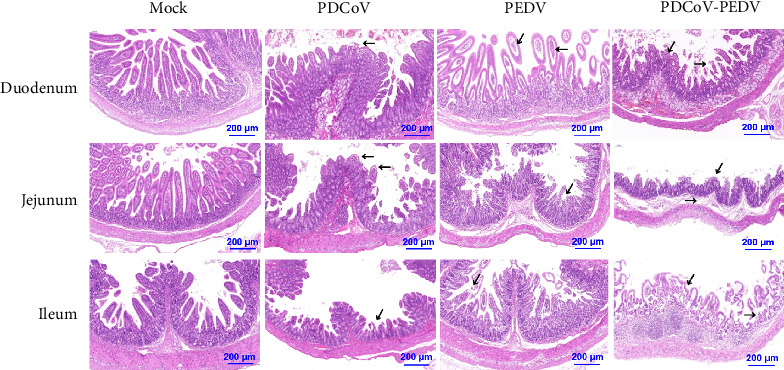# Correction to “Co-Infection of Porcine Epidemic Diarrhoea Virus and Porcine Deltacoronavirus Enhances the Disease Severity in Piglets”

**DOI:** 10.1155/tbed/9782932

**Published:** 2025-09-27

H. Zhang, F. Han, X. Shu et al., “Co-Infection of Porcine Epidemic Diarrhoea Virus and Porcine Deltacoronavirus Enhances the Disease Severity in Piglets,” *Transboundary and Emerging Diseases*, 2021, vol. 69: 1715–1726, https://onlinelibrary.wiley.com/doi/10.1111/tbed.14144.

In the article, there is an error in Figure 1b, introduced during the preparation of the figure. Specifically, the PDCoV and PEDV treated Ileum tissue panels contain repeated elements. The correct Figure 1b is shown below:

We apologise for this error.

## Figures and Tables

**Figure 1:**